# Lifesaving pericardiocentesis due to purulent pericarditis with growth of Gram-negative rods in an immune-competent Inuit male

**DOI:** 10.1186/s12245-014-0021-8

**Published:** 2014-07-09

**Authors:** Carl Frederik Brandt Simonÿ, Mikkel Malham, Jacob Kanstrup, Piotr Wojtek, Folmer Lynggaard, Stig Andersen

**Affiliations:** 1 Department of Internal Medicine, Queen Ingrid’s Hospital, Nuuk 3900, Greenland; 2 Department of Anaesthesiology, Queen Ingrid’s Hospital, Nuuk 3900, Greenland; 3 Department of Radiology, Queen Ingrid’s Hospital, Nuuk 3900, Greenland; 4 Department of Geriatric Medicine, Aalborg University Hospital, Aalborg 9000, Denmark; 5 Arctic Health Research Centre, Institute of Clinical Medicine, Aalborg University Hospital, Aalborg 9210, Denmark

**Keywords:** Rural echocardiography, Pericarditis, Pericardiocentesis, Tamponade, Proteus species, Escherichia coli

## Abstract

Polymicrobial Gram-negative pericarditis is a rare entity in the immune-competent patient, and purulent pericarditis due to bacteria complicated by tamponade is a life-threatening condition with high mortality rates. A prompt diagnosis and treatment is, as in this case, lifesaving and facilitated by echocardiography but is not the case in rural areas in common. Change in the infectious aetiology indicates broad-spectrum antibiotics. We describe a case of purulent pericarditis causing cardiac tamponade due to haematogenous spread of *Escherichia coli (E. coli)* and *Proteus* species in an immune-competent Inuit male treated with pericardiocentesis.

## Background

Purulent pericarditis (PP), caused by bacteria and complicated by tamponade, is a rare and life-threatening condition with mortality rates ranging from 30% to 75% [1]-[4]. A prompt diagnosis and treatment is lifesaving and has been facilitated by the availability of echocardiography, which is now the standard equipment in most emergency rooms in the western world. However, this is not the case in many rural areas [5]. Treatment with broad-spectrum antibiotics is indicated as there has been a change in infectious aetiology, and the number of polymicrobial infections rises [3],[4],[6]. We report a case of purulent pericarditis causing cardiac tamponade due to haematogenous spread of *Escherichia coli* and *Proteus* species in an immune-competent Inuit male. This was diagnosed in rural North Greenland where also emergency pericardiocentesis was performed.

## Case presentation

A 53-year-old man presented with abdominal pain at the first visit to the local nursing station in a rural settlement in North Greenland in August 2012. He was pale and shivering and had a temperature of 37.6°C, blood pressure of 105/65 mmHg and a heart rate of 92/min. He improved on morphine injection. The following day, he had persistent abdominal pain, a low blood pressure of 60/40 mmHg, heart rate of 70, normal electrocardiogram (ECG) but was described as well circulated and was dismissed with antiemetics without further notice.

At the second visit in September 2012, he reported a weight loss of 10 kg, fatigue, cough, and nausea. He was sallow, shivering, and sweating. Laboratory tests showed haemoglobin of 5.6 mmol/L, raised ESR of 110 mm/hour, CRP of 74 mg/L (reference range, 0 to 8 mg/L), normal liver function tests, and QuantiFeron negative. Chest X-ray was normal. He was transferred to the local hospital in North Greenland where a weight loss of 8.5 kg was confirmed. The local hospital referred him for further evaluation at the main hospital in the capital city Nuuk. He was sent home awaiting admission in Nuuk.

**Figure 1:**
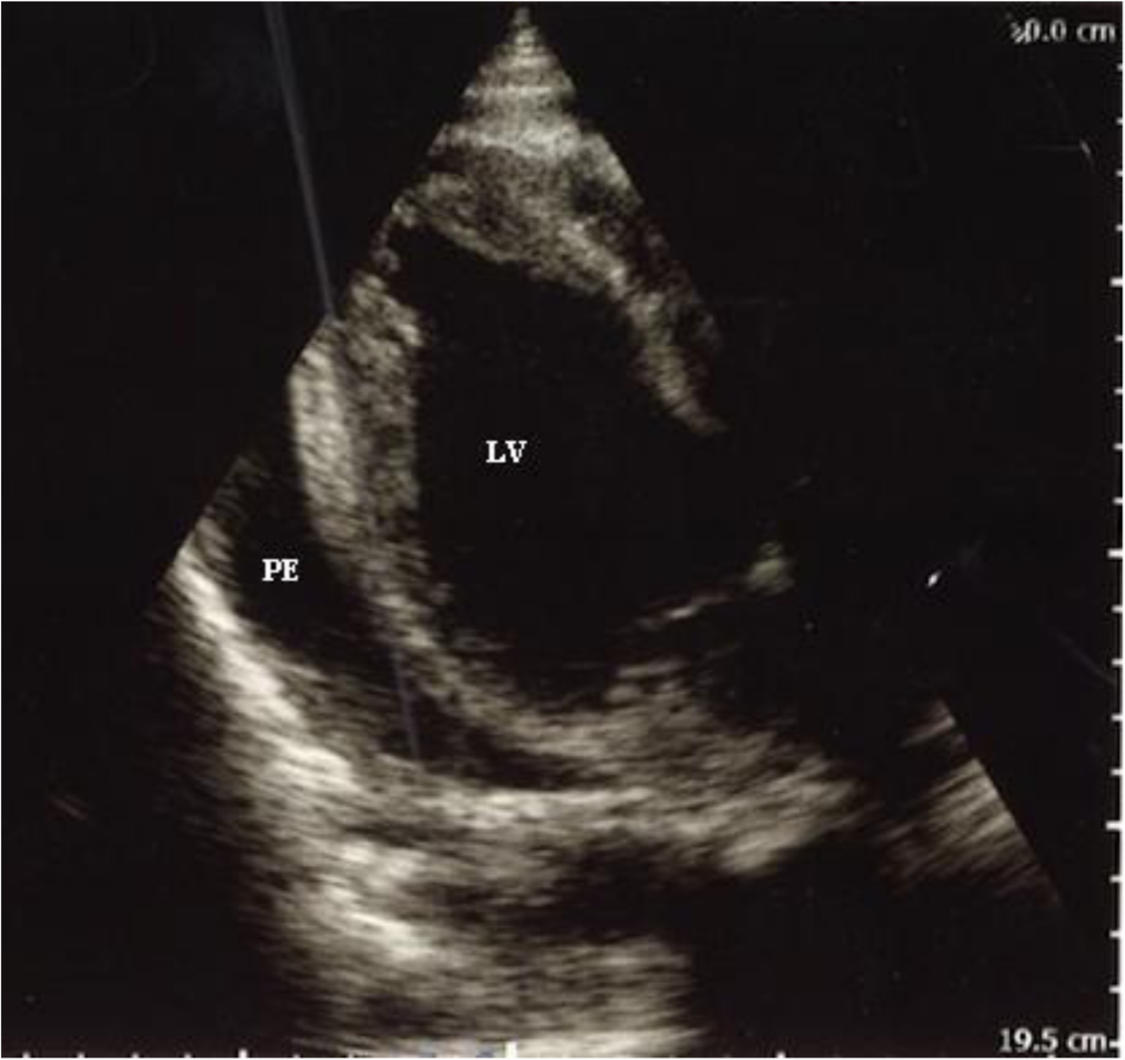
**Echocardiography (parasternal long axis view) prior to the initial pericardiocentesis in rural North Greenland.** The pericardial effusion (PE) is seen. LV, left ventricle.

At the third visit to the local nursing station in October 2012, he presented with abdominal pain and melena. His condition deteriorated, and antibiotics (intravenous ceftriaxone) were initiated. The patient was then transferred to the local hospital in North Greenland for stabilization prior to transport to the main hospital in Nuuk. His hands and feet were cold; he was pale and sweating and complained of neck pain. Blood pressure was 63/50 mmHg, and heart rate was 60/min. Laboratory findings were as follows: haemoglobin 4.8 mmol/L, white blood cell count 23.4 × 10^9^/L, CRP above 160 mg/L, creatinine 285 μmol/L (reference range, 61 to 132 μmol/L), and a normal blood glucose. Chest X-ray was normal except for an elevated right diaphragm. ECG was normal. He developed tachypnea, acrocyanosis, immeasurable blood pressure, and marked jugular vein distension. An echocardiography revealed pericardial fluid (Figure 1).

Pericardiocentesis was performed, and 100 mL purulent pericardial fluid was drained. No culture was done at this point. Upon slight clinical improvement, the patient was transported 500 km south to Queen Ingrid's Hospital in the capital city, Nuuk.

**Figure 2:**
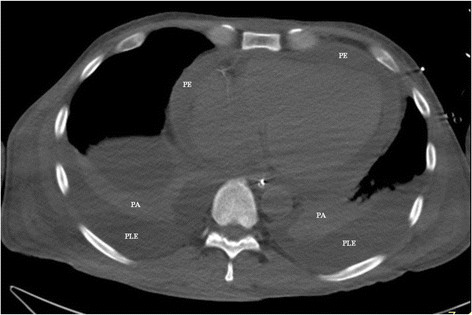
**CT scanning of the thorax without contrast.** The pericardial effusion (PE) surrounding the heart is about 15 mm thick. PLE, bilateral pleural effusions; PA, pulmonary atelectasis.

In Nuuk, the patient presented with multiple organ failure and was stuporous and arterial blood gas showed pH of 6.98, base excess of −18 mmol/L and pCO_2_ 7.1 kPa. The patient was sedated and put on mechanical ventilation. Pericardiocentesis drained a further 500 mL of purulent fluid with a subsequent slight rise in blood pressure. Marked pericardial fluid persisted (Figure 2).

Intrapericardial fibrinolysis was performed, and subsequently, an additional 1,000 mL of purulent fluid was drained. After pericardiocentesis, blood pressure stabilized at 120/65 mmHg supported by inotropy that was reduced from day 5 in Nuuk. Still, the patient was anuric and dialysis was started on day 3 in Nuuk.

Culture from pericardial fluid showed *E. coli* and *Proteus* species. Blood cultures were collected 3 days after initiation of antibiotics and were without bacterial growth. Human immunodeficiency virus (HIV), hepatitis B and C, QuantiFeron and treponema pallidum antibodies were negative. There were no tumour cells, acid-fast rods or spirochaetas in neither the pericardial fluid nor in the pleural fluid. Computer tomography (CT) of the chest and abdomen was normal apart from the pericardial fluid, pleural effusion and atelectasis. Gastroscopy and thoracotomy performed in Denmark did not provide an explanation for transmission of bacteria. Radiologic examination of the gastrointestinal tract with contrast medium showed no passage to the pericardium.

The patient recovered and was dismissed to his home settlement after 3 months.

## Discussion

PP is a life-threatening condition, which can be complicated with tamponade. It is now rare and comprises less than 1% of patients presenting with acute pericarditis [2]. This is most likely due to the widespread use of antibiotics that reduces the risk of complications previously seen subsequent to common infections [1]. However, quality of care in rural areas may increase the risk of infections that develop complications.

We present a case of PP with cardiac tamponade due to 1,500 ml of purulent fluid in the pericardial sack infected with *E. coli* and *Proteus* sp. in an immune-competent Inuit male living in a rural settlement in North Greenland. This is unusual for several reasons.

First, bacteria most commonly seen in PP are *streptococcus* and *staphylococcus*[3],[4]. Culture of the pericardial fluid grew *E. coli* and *Proteus* species. These are common inhabitants of the gastrointestinal tract and are known to cause infections of the genitourinary tract but have not previously been reported in the pericardial fluid. Treatment with antibiotics may promote *Proteus* growth. This was, however, not the case with our patient. The combination with *E. coli* and *Proteus* species suggests an abdominal origin [4]. Yet, the patient did not have signs of urinary tract infection or gastrointestinal complaints apart from an episode of abdominal pain 2 months prior to admission. Unfortunately, a urine culture was not done.

Pericardial infections with more than one bacterium are less common compared to pericarditis due to single organisms. Still, detection of more than one bacterium may have increased over the last decades [4]. Thus, Sawaya and colleagues described a case of cardiac tamponade due to the two Gram-negative organisms, *Citrobacter diversus* and *Proteus mirabilis*. These are inhabitants of the gastrointestinal flora and are known to cause infections of the genitourinary tract. Furthermore, pericarditis due to two or more bacteria is more frequently caused by Gram-negative bacilli [4]. This is in keeping with the findings in our case.

Second, PP usually develops from an evident site of infection. This is most often due to adhesion and direct extension from pneumonia, empyema, subphrenic abscess or direct perforation from a cancer or trauma [3]. Despite a thorough workup, no site of infection was identified. Culture of the pericardial fluid grew *E. coli* and *Proteus* species sensitive to ceftriaxone, on which the patient started before transport to the local hospital. Blood cultures were taken 3 days after the initiation of antibiotic therapy due to logistics in North Greenland and were, as could be expected, negative. However, no other route of inoculation was found and haematogenous dissemination is most likely.

Third, individuals prone to PP are patients with underlying disease that predisposes to immunodeficiency and infection [2],[3],[6]. This was not the case with our patient. He did not host HIV or show signs of other predisposing factors or immunologic disorders.

Fourth, *Mycobacterium tuberculosis* is a common cause of pericarditis in other populations [2]. Also, tuberculosis is a relatively common infection in Greenland in general [7]–[9]. Still, this patient had no signs of previous or present tuberculosis in neither the pericardial fluid nor elsewhere.

Intrapericardial fibrinolysis has been suggested as a less invasive method for prevention of persistent pericarditis and constrictive pericarditis, as fibrin formation is a cornerstone in pathogenesis of these conditions [10]. In this case, pericardial fluid persisted after drainage of 500 ml of purulent fluid from the pericardium. Another 1,000 ml of purulent fluid was evacuated through the pericardial drain only after repeated fibrinolysis of the pericardium. This confirmed loculation that prevented drainage of the persistent purulent discharge and may have contributed to the survival of this patient.

Rural nursing station staff comprises a helper with 3 months of training at the local hospital, a nursing station attendant and, in addition, a nurse in the larger settlements. Furthermore, the small rural hospital (4 doctors, 16 beds) has limited equipment. Some blood tests are analysed locally, and X-ray and ultrasound equipment is available. Still, lifesaving echocardiography was performed for diagnosis and initial pericardiocentesis with the basic equipment. This was crucial as there are no roads, and all transport is either by boat or by air. The 500 km to the main hospital in Nuuk required aviation transport with an obligatory low oxygen availability due to the lower pressure during air transport. Acrocyanosis and tachypnea suggested low of oxygenating capacity probably due to low cardiac output. The slight improvement after pericardiocentesis allowed for transport to the hospital in the capital Nuuk.

Computerized tomography is valuable in the identification of an origin of infection when this is localized closely to the heart [4]. However, neither CT scan nor gastroscopy gave any clue to an origin of the infection in our case.

## Conclusion

In conclusion, complications subsequent to infections may be more frequent in rural areas, and the diagnosis of PP with tamponade may be suspected from clinical findings and is supported by echocardiography. The case illustrates the importance of the availability of echocardiography in rural health care as a simple diagnostic tool of a life-threatening disease that may otherwise be overlooked. Furthermore, it provides the possibility of lifesaving emergency pericardiocentesis.

This case also shows the possibility of PP to develop over a long period of time, and it illustrates the potential for atypical transmission of infection to the pericardium in an otherwise healthy individual. Finally, ours and other cases lend support to early broad-spectrum antibiotic treatment, with agents covering both Gram-positive and Gram-negative organisms and later narrowing of therapy once the causal agent is identified.

## Consent

Obtaining a consent is not possible. Patient is not identifiable in the case report.

## Ethic statement

The study was approved by Queen Ingrid’s Hospital at Nuuk and has been performed in accordance with the ethical standards as laid down in Declaration of Helsinki.

## Competing interests

The authors declare that they have no competing interests.

## Authors’ contributions

CFS participated in the conception of idea, data collection, analysis and interpretation of data and writing of the manuscript. MM participated in the conception of idea, data collection, interpretation of data and writing of the manuscript. JK, PW and FL participated in data collection, interpretation of data and reviewing of the manuscript. SA participated in the conception of idea, analysis and interpretation of data and writing of the manuscript. All authors read and approved the final manuscript.

## Funding sources

None of the authors have received any fees for this manuscript.
